# Advancing glassy carbon microelectrode arrays for neurochemical sensing: impact of double pyrolysis on structure and function

**DOI:** 10.3389/fbioe.2025.1642063

**Published:** 2025-09-19

**Authors:** Sarah Catherine Sellen, Umisha Siwakoti, Ashok Sigdel, Bicky Jaiswal, Sandra Zivanovic, Elisa Castagnola

**Affiliations:** ^1^ Department of Electrical Engineering, Louisiana Tech University, Ruston, LA, United States; ^2^ Department of Biomedical Engineering, Louisiana Tech University, Ruston, LA, United States; ^3^ Institute for Micromanufacturing, Louisiana Tech University, Ruston, LA, United States

**Keywords:** glassy carbon, microelectrode array, pyrolysis, sheet resistance, fast scan cyclic voltammetry, serotonin, dopamine

## Abstract

Advancing neural interfaces requires implantable devices capable of long-term electrical and chemical monitoring. “All”-glassy carbon (GC) microelectrode arrays (MEAs), in which both electrodes and interconnects are formed from homogeneous GC layer, offer integrated chemical sensing and electrophysiological recording, while enhancing electrochemical durability by eliminating metal components. To guide the development of high-resolution, double-layer “all”-GC MEAs for higher-density architectures, this study systematically investigates GC as both an interconnect and neurochemical sensing material, with particular focus on the effects of double pyrolysis on structural integrity, interconnect resistance, and microelectrode performance. Sheet resistance was analyzed across films of varying thicknesses, and interconnect geometry was evaluated. Raman spectroscopy and X-ray diffraction characterized graphitization and crystallinity, while fast-scan cyclic voltammetry (FSCV) assessed dopamine and serotonin detection. A 48% reduction in the thickness of once-pyrolyzed GC corresponds to a 63% increase in its sheet resistance. A double pyrolyzed GC trace has about 50% higher sheet resistance than a single-pyrolyzed GC trace of the same thickness. Double pyrolysis caused approximately 20% shrinkage in the GC layer. Compared to Cr/Au/Pt traces, GC interconnects had higher resistance at 1–3 µm widths but approached metal-like performance at 5–10 µm. Importantly, the second pyrolysis cycle preserved structural integrity and FSCV sensitivity. These analyses advance our understanding of GC’s electrical and sensing properties, providing critical insights for optimizing compact multilayer devices in next-generation “all”-GC-MEAs.

## 1 Introduction

Neural communication relies on both electrical and chemical signaling, with neurotransmitters operating across various timescales and interacting in complex brain functions. To study these mechanisms more effectively, implantable devices capable of long-term, multimodal monitoring of both electrical activity and neurotransmitter dynamics are essential.

Implantable microelectrode arrays (MEAs) have become indispensable for recording neurophysiological activity with single-neuron resolution across multiple brain regions and depths (Gong et al., 2025; Tanwar et al., 2022; Zhuang et al., 2014; G et al., 2015). Traditional MEAs, typically incorporating metal electrodes and interconnects, such as gold, platinum or iridium, on either rigid [silicon-based (Gong et al., 2025; Tanwar et al., 2022; Zhuang et al., 2014; G et al., 2015)] or flexible [polymer-based (Luan et al., 2020; Luan et al., 2017; Wei et al., 2018; Zhao et al., 2023)] substrates, offer excellent performance for electrophysiological recordings. However, they lack the chemical sensitivity required to detect key electroactive neurotransmitters, including dopamine (DA) and serotonin (5-HT), thereby limiting their capability in multimodal applications. A critical step toward enhancing flexible MEAs is the development of electrode and interconnect materials that combine electrical conductivity, chemical sensitivity, and electrochemical stability. Carbon offers a compelling solution, exhibiting high biocompatibility, rapid electron transfer kinetics, a wide electrochemical window, and superior stability, along with excellent sensitivity to redox-active neurotransmitters (Rafi and Zestos, 2021; Cao et al., 2019; Castag et al., 2010). Carbon-based electrodes are also highly compatible with fast-scan cyclic voltammetry (FSCV), enabling sub-second resolution neurochemical sensing (Puthongkham and Venton, 2020; Roberts and Sombers, 2018).

Recent advances have introduced innovative fabrication strategies incorporating diamond (Fan et al., 2020; Fan et al., 2023), glassy carbon (GC) (Castagnola et al., 2022; Vahidi et al., 2020; Castagnola et al., 2018; Castagnola et al., 2021; Castagnola et al., 2024), and thermally stabilized graphene microelectrodes (Qi et al., 2025) into flexible neural probes. These platforms are compatible with FSCV and have demonstrated reliable performance in neural recording applications (Fan et al., 2020; Castagnola et al., 2018; Castagnola et al., 2021; Qi et al., 2025). Nevertheless, most of these devices still rely on metal interconnects alongside carbon electrodes, and the adhesion between metal and carbon layers raises concerns about long-term reliability under the electrical and mechanical stresses associated with chronic use, particularly during high-frequency FSCV.

A notable advancement is the development of GC-MEAs in which both electrodes and interconnects are made entirely of a homogeneous GC layer (“all”-GC-MEAs) (Faul et al., 2024; Nimbalkar et al., 2018). The first prototype, developed by the Kassegne group, demonstrated exceptional electrochemical durability, enduring over 3.5 billion charge-balanced current pulses without failure (Nimbalkar et al., 2018). By eliminating adhesion issues between dissimilar conductive layers, such as metal interconnects and carbon electrodes, this design enhances long-term stability under prolonged electrical and mechanical stress. Building on this approach, “all”-GC-MEAs have been fabricated on flexible substrates with GC trace widths reduced to 3 µm (Faul et al., 2024; Siwakoti et al., 2025a), while retaining FSCV functionality and effective 5-HT sensing (Faul et al., 2024). However, further optimization is required to improve process control and scalability, as well as to fully assess the conductivity and long-term reliability of micrometer-wide GC interconnects in highly miniaturized architectures.

This study presents a comprehensive investigation of GC as both an interconnect and neurochemical sensing material to guide the development of high-resolution, double-layer “all”-GC-MEAs for higher-density architectures. We examine (a) how GC sheet resistance varies with film thickness and (b) how interconnect geometry—trace width and length—affects overall resistance across different GC thicknesses, with the goal of optimizing layouts for miniaturized, high-density devices. Resistance values are benchmarked against metal traces of equivalent dimensions to contextualize GC’s electrical performance. We also evaluate the effects of repeated pyrolysis on GC interconnect resistance, structural integrity, and microelectrode sensing performance, as the ability to undergo a second pyrolysis without detrimental effects is critical for double-layer fabrication. Raman spectroscopy and X-ray diffraction (XRD) characterize graphitization, defect density, and crystallinity, while FSCV assesses 5-HT and DA detection capabilities. Together, these analyses provide comprehensive insight into GC’s electrical, structural, and sensing properties, informing the optimization of next-generation “all”-GC-MEAs designs.

## 2 Materials and methods

### 2.1 Pyrolysis process

Pyrolysis was carried out following previously reported procedures from our lab (Faul et al., 2024; Siwakoti et al., 2025b). First, four-in Si wafers with a 1 µm thick SiO_2_ layer (University Wafer Inc., Boston, MA, United States) were cleaned with acetone, isopropanol, and deionized water (DI) sequentially. The wafers were then dried with an N_2_ spray gun and heated on a hot plate at 200 °C for 5 min. The cleaned wafers were spin-coated with SU-8 3035 (Kayaku Advanced Materials, Westborough, MA, United States) for 1 min at 2000 rpm, 3000 rpm and 4000 rpm, respectively, to achieve different thickness. The SU-8 coated wafers were then soft baked at 65 °C for 3 min and 95 °C for 7 min. Then, the wafers were exposed using a custom-made photomask and a MA/BA6 Mask/Bond Aligner (Süss MicroTec, Garching, Germany) with a dose of 350 mJ/cm^2.^ After exposure, the wafers were post-baked at 65 °C for 1 min and 95 °C for 5 min, then developed using SU-8 developer (Kayaku Advanced Materials, Westborough, MA, United States) for 60 s and cleaned with isopropanol and DI water. The patterned SU-8 was hard-baked at 150 °C for 5 min then at 200 °C for 5 min and allowed to cool down below 65 °C. The first pyrolysis of the negative SU-8 resist was performed in a high-temperature split tube furnace (STF 1200 Tube Furnace, Across International, Livingston, NJ, United States). The sample was heated to 900 °C with a temperature ramp-up at a rate of 5 °C/min, then maintained at 900 °C under 15 standard cubic centimeters per minute (sccm) N_2_ (Airgas, Radnor Township, PA, United States) at 0.8 Torr for 60 min. The sample was then slowly cooled to room temperature at 3 °C/min. The wafers were then cut in half. One-half of the wafers then underwent a second pyrolysis process that was identical to the first pyrolysis process to produce the double pyrolysis GC.

### 2.2 Sheet resistance measurements

Sheet resistance was measured for each of the 3 GC thicknesses on both single- and double-pyrolyzed wafer halves using a Jandel RM3000 four-point probe system (Jandel, Eagle, ID, United States). For each electrode, six measurements were taken to ensure consistency across positions and probe orientations: three with the probe oriented vertically and three horizontally. Sheet resistance measurements were conducted at the center, as well as the left and right edges, of each 6 mm × 10 mm rectangular GC electrode. This electrode size was selected to accommodate the four-point probe stage. All electrodes were patterned and pyrolyzed under the same conditions used for fabricating the GC-MEA features, ensuring consistency across the tested and functional devices. In all cases, the four probes maintained full contact with the GC surface. A constant current of 1 mA was applied during all measurements.

### 2.3 Raman spectroscopy

Raman spectroscopy measurements were performed using the Horiba XploRA Plus Raman microscope (Horiba, Piscataway, NJ, United States). A 532 nm laser was used with a grating of 1200 gr/mm on the GC through a ×100 objective. An average of five 10-s acquisitions were used, and cosmic ray spikes were removed in LabSpec six software (Horiba, Piscataway, NJ, United States).

### 2.4 XRD diffraction analysis

The XRD data were captured with the Bruker D8 diffractometer (Bruker, Billerica, MA, United States). The X-ray source is copper with a wavelength of approximately 1.54 Å. The total runtime for the sample was 1 h and 20 min for each of the 3 regions scanned.

### 2.5 Morphological and chemical characterization

Scanning electron microscopy (SEM) and elemental analysis of surfaces in field-emission SEM were performed using energy-dispersive spectrometry (EDS) to identify and quantify all present elements using a HITACHI S-4800 field-emission SEM with a Bruker (Xflash 6160) EDS attachment (HITACHI Global, Irvine, CA, United States). High resolution optical imaging was performed using a VK-X150 3D scanning confocal microscope (Keyence America, Itasca, IL, United States).

### 2.6 Hybrid GC-MEA fabrication

The hybrid GC-MEA fabrication follows the procedure established in (Castagnola et al., 2022; Castagnola et al., 2023a). The fabricated probes contained 5–8 microelectrodes along a 100–140 µm-wide shank, with circular (50 µm diameter, ∼1,963 μm^2^) or oval (75 μm × 35 μm, ∼2,062 μm^2^) electroactive areas used for chemical measurements. First, a 4-inch silicon wafer with a 1 μm-thick thermal SiO_2_ layer (University Wafer Inc., Boston, MA, United States) was cleaned using sequential rinses in acetone, isopropanol, and deionized (DI) water, followed by drying with a nitrogen (N_2_) spray gun. The wafer was further dried on a hot plate at 150 °C for 5 min. Surface activation was then performed using O_2_ plasma in a reactive ion etcher (RIE, MICRO-RIE 800, Technics Inc., Anaheim, CA, United States) operated at 300 mTorr and 150 W for 60 s. Following plasma treatment, SU-8 3035 (Kayaku Advanced Materials, Westborough, MA, United States) was spin-coated at 3000 rpm for 1 min. The coated wafer was soft-baked at 65 °C for 5 min and then at 95 °C for 5 min. Photolithographic patterning was performed using a custom-designed photomask and a MA/BA6 Mask/Bond Aligner (Süss MicroTec, Garching, Germany) with an exposure dose of 350 mJ/cm^2^. After exposure, the wafer underwent a post-exposure bake at 65 °C for 1 min and 95 °C for 3 min. Development was carried out in SU-8 developer (Kayaku Advanced Materials) for 1 min, followed by rinsing in isopropanol and DI water. The patterned SU-8 was subsequently hard-baked at 200 °C and 150 °C for 5 min each, then allowed to cool below 65 °C. Single or double pyrolysis was performed using a high-temperature split tube furnace, as previously described in Section 2.1. Following pyrolysis, the wafer was cleaned again using acetone, isopropanol, and DI water, dried with a N_2_ spray gun, and treated with oxygen plasma using RIE. The cleaned wafer was then spin-coated with SU-8 5 (Kayaku Advanced Materials, Westborough, MA, United States) at 3000 rpm for 1 min, followed by a soft bake at 65 °C for 3 min and 95 °C for 5 min. This SU-8 layer was subsequently patterned to define the insulation layer using a UV exposure dose of 200 mJ/cm^2^. After exposure, a post-exposure bake was performed at 65 °C for 1 min and 95 °C for 3 min. The wafer was then developed in SU-8 developer (Kayaku Advanced Materials), rinsed with isopropanol and DI water. Finally, the patterned wafer was hard-baked as previously described. In the next step, it was spin-coated with APOL-LO 3204 (KemLab, Woburn, MA, United States) at 3000 rpm for 1 min and soft-baked at 110 °C for 1 min. The APOL-LO 3204 layer was patterned using a dose of 145 mJ/cm^2^, to create a sacrificial mask for the subsequent metal lift-off. After a post-bake at 110 °C for 1 min, the wafer was developed using TMAH developer (KemLab). Subsequently, the metal layers were deposited onto the wafer by sputtering: 15 nm of chromium, 85 nm of gold, and 20 nm of platinum. The wafer was immersed in acetone for the lift-off process, then cleaned with isopropanol to remove any residual debris. The wafer was then cleaned again with acetone, isopropanol, and DI water, dried with an N_2_ spray gun, and spin-coated with SU-8 5 (Kayaku Advanced Materials, Westborough, MA, United States) at 1000 rpm for 1 min. It was then soft-baked at 65 °C for 5 min and 95 °C for another 5 min. The SU-8 layer was patterned using a dose of 200 mJ/cm^2^ to define the insulation layer. A post-bake followed at 65 °C for 2 min and 95 °C for 3 min, and the wafer was developed using SU-8 developer. Finally, the patterned wafer was cleaned with isopropanol and DI water, and hard-baked. The microelectrode arrays (MEAs) were released from the wafer using a 1:7 buffered oxide etchant in an acid hood for 4–6 h.

### 2.7 Fast scan cyclic voltammetry

The sensitivity and stability tests were conducted using hybrid GC-MEAs (glassy carbon electrodes and metal interconnections) to compare the sensing performance of GC electrodes carbonized once *versus* twice. The FSCV measurements of 5-HT and DA were performed using a FSCV Wave Neuro potentiostat connected to a Flow Cell System (Pine Research, Durham, NC, United States) to assess the sensitivity of the GC microelectrodes. Data acquisition and analysis were conducted using HDCV software (University of North Carolina at Chapel Hill, Chapel Hill, NC, United States). For 5-HT detection, a modified N-shaped waveform (0.2 V → 1.3 V → −0.1 V → 0.2 V) was applied at a scan rate of 1000 V/s and a frequency of 10 Hz, following procedures previously described (Faul et al., 2024). For DA detection, a triangular waveform (−0.4 V → 1.0 V → −0.4 V vs. Ag/AgCl) was used at a scan rate of 400 V/s, consistent with previously reported protocols (Castagnola et al., 2021). 5-HT and DA were identified by inspection of the background-subtracted cyclic voltammograms. Electrodes were tested with 1 μM 5-HT or DA prepared in 1× phosphate-buffered saline (PBS, composition: 11.9 mM Na_2_HPO_4_ and KH_2_PO_4_, 137mM NaCl, 2.7 mM KCl, pH 7.4), introduced via bolus injection. The Flow Cell System was continuously perfused with 1× PBS at a rate of 60 mL/h using a syringe pump. To assess the stability of the background current over time, we applied the FSCV waveform used for DA detection at 50 Hz to GC microelectrodes pyrolyzed once or twice in PBS for 30 consecutive hours. Prior to testing, the electrodes were preconditioned by cycling at 60 Hz for 30 min using the same waveform, similar to preconditioning protocols reported for carbon fibers (Takmakov et al., 2010; Meunier et al., 2017).

### 2.8 Statistical analysis

Statistical analyses were conducted using Origin 2024 software (OriginLab Corp., Northampton, MA, United States). Unpaired two-sample t-test at a 95% confidence level was used to compare the D/G ratio of GC before and after the second pyrolysis cycle. One-way ANOVA with Bonferroni post-tests was used to compare changes in the sensitivity of GC pyrolyzed once and twice to 5-HT. Significance was determined at *p* < 0.05. To evaluate the chemical composition of GC after one or two pyrolysis cycles, a two-sample t-test was performed. Differences were considered statistically significant at *p* < 0.05.

## 3 Results and discussions

### 3.1 Electrical properties of single pyrolyzed glassy carbon

Developing miniaturized devices in which both the electrodes and interconnects are made entirely of GC will be transformative in extending the lifetime of implantable devices for chronic neural interfaces. This “all”-GC approach eliminates concerns due to adhesion issues between dissimilar conductive layers, such as metal interconnects and carbon electrodes, thereby removing potential sources of mechanical or electrical failure under prolonged electrical stimulation and mechanical stress. While GC is well-established as a neurochemical sensing material, its suitability as an interconnect requires deeper characterization. We therefore systematically examined how GC sheet resistance varies with film thickness and how interconnect width and length affect total resistance. These results aim to inform the optimization of GC interconnect geometry for scalable, high-density device fabrication.

We successfully pyrolyzed GC samples with varying thicknesses using SU-8 precursors of different initial thicknesses. During pyrolysis, the hard-baked SU-8 shrank in average by 74.1% in thickness, similar to previously reported (Faul et al., 2024), 2.6% in length and 4.8% in width, a factor that must be considered when selecting SU-8 viscosity and spin-coating parameters.

The sheet resistance was measured on multiple samples of GC with three different thicknesses, each prepared using one or two pyrolysis cycles. Table 1 summarizes the sheet resistance measurements of GC samples with three different thicknesses, each prepared using a single pyrolysis cycle, and compares them with metal samples of the same dimensions.

**TABLE 1:** Sheet resistance of single-pyrolyzed glassy carbon at three thicknesses compared to metal.

Material	Thickness (µm) (*n* = 10)	Sheet resistance *ρ* *_s_* (Ω/square) (*n* = 14)
GC	3.81 ± 0.12	48.78 ± 4.11
GC	2.81 ± 0.085	62.02 ± 3.91
GC	1.98 ± 0.055	79.53 ± 4.72
Metal (Cr, Au, Pt)	0.12	2.44 ± 0.39

The metal samples were fabricated via lift-off patterning of a sputtered multilayer stack consisting of 15 nm chromium (Cr), 20 nm gold (Au), and 85 nm platinum (Pt). To confirm the consistency of sheet resistance across the wafer and with different pin probe orientations, six measurements were performed on each of the fourteen electrodes per thickness. Three measurements were taken with vertically oriented probes and three with horizontally oriented probes, positioned at the left edge, center, and right edge of each GC and metal electrode. The results showed consistent sheet resistance across all measurement sites, indicating uniform electrical properties for each GC thickness. As anticipated, we observed a decrease in sheet resistance with an increase in GC thickness. Specifically, the sheet resistance increased from 48.78 ± 4.11 Ω/square for GC with a thickness of 3.81 ± 0.12 μm to 79.53 ± 4.72 Ω/square for GC with a thickness of 1.98 ± 0.055 μm. This 48% decrease in thickness leads to a 63% increase in sheet resistance.

Despite limited literature on the sheet resistance of GC films at microelectrode-scale pyrolysis thicknesses, laser-induced graphene (LIG) offers a relevant benchmark due to its structurally similar carbon framework and tunable conductivity (Li et al., 2022; Nam et al., 2022; Abdulhafez et al., 2021). Derived from commercially available or engineered polyimides (PIs) via laser irradiation, LIG has emerged as a promising method for fabricating carbon-based electrodes and interconnections. Its ability to form large-area, porous graphene with controllable pore density makes it especially suitable for advanced electrode designs (Li et al., 2022; Nam et al., 2022; Abdulhafez et al., 2021). Reported sheet resistance values for LIG range widely—from approximately 29–2476 Ω/square—depending on laser power and fabrication parameters (Kim et al., 2025). Notably, LIG formed under 200 mW irradiation has exhibited particularly high conductivity, with sheet resistance values as low as 30–50 Ω/square at film thicknesses of 10–20 µm (Kim et al., 2025).

Although LIG and pyrolyzed GC differ significantly in fabrication methods, microstructure, and crystallinity, these values offer a comparative benchmark. Our pyrolyzed GC films exhibit comparable sheet resistance performance while achieving these values with 3–30 times lower film thicknesses, underscoring their potential advantages for high-density, miniaturized interconnects in implantable devices. LIG offers rapid prototyping and material versatility, but its resolution is limited by laser spot size (Lee et al., 2023), making sub-50–100 µm features difficult. Typical LIG line widths (50–100 µm) exceed those of photolithography, restricting electrode density and preventing microelectrodes small enough for single-unit recordings.

Compared to noble metals, the current standard for electrodes and interconnections in neural probes, which are typically patterned with a thickness of a few hundred nanometers (Luan et al., 2017), GC exhibits a sheet resistance approximately one order of magnitude higher, even though it presents a much higher thickness, as reported in Table 1. However, it offers several distinct advantages that make it highly suitable for neural interface applications. Among its most compelling properties is its exceptional electrochemical stability in physiological environments, which supports long-term functionality without significant degradation (Nimbalkar et al., 2018). Furthermore, GC’s capability to detect electroactive neurotransmitters using sub-second resolution FSCV (Castagnola et al., 2022; Castagnola et al., 2021) enables multimodal recording from a single device (Castagnola et al., 2021; Castagnola et al., 2024), making it highly attractive for advanced multimodal platforms.

**FIGURE 1:**
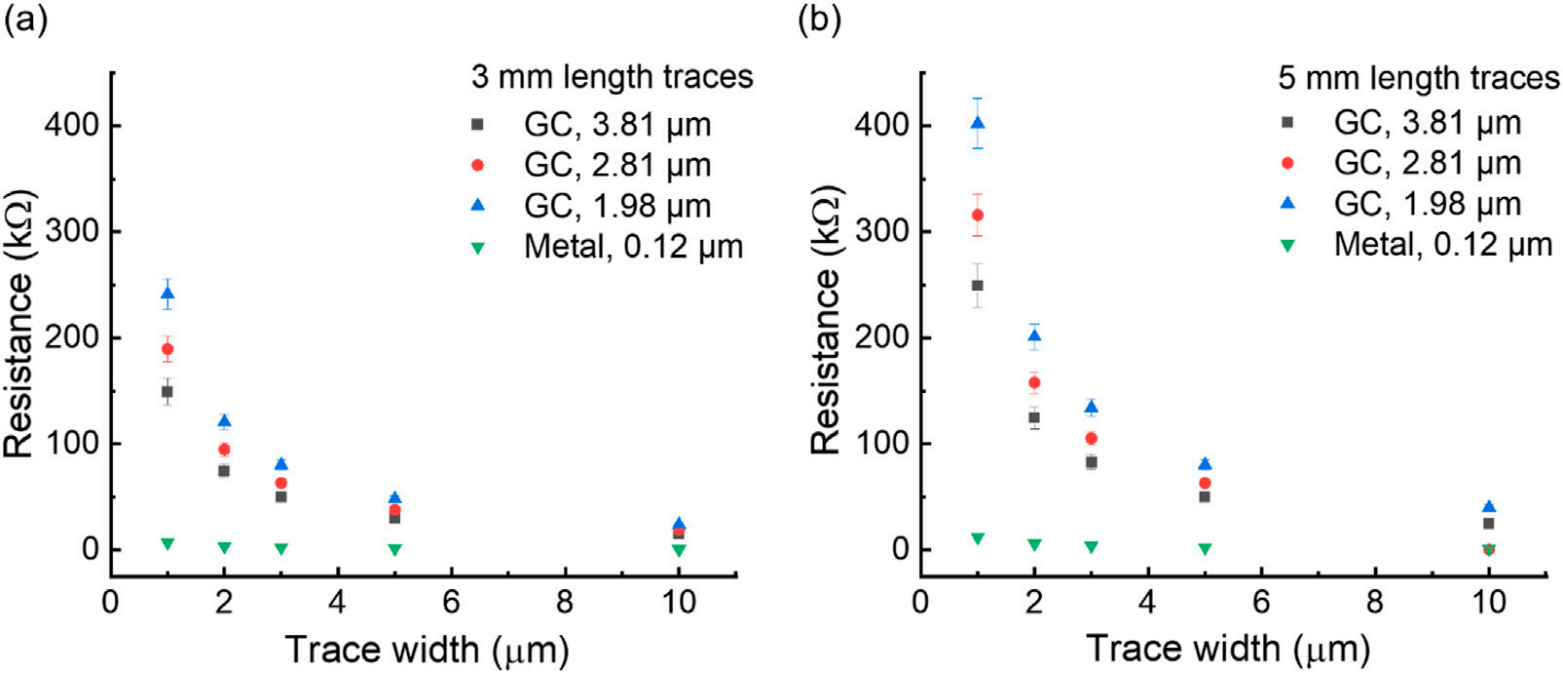
Resistance of pyrolyzed-once GC with respect to trace width for 3.81μm, 2.81μm, and 1.98 μm thicknesses for 3 mm length **(a)** and 5 mm length **(b)** traces, in comparison with metal traces of the same width and 120 nm thickness (Cr 15 nm, Au 20 nm, Pt 85 nm).

Understanding the extent to which GC structures can be miniaturized in terms of thickness and width is also critical, as this could enable the development of higher-density, smaller-footprint neural interfaces—ultimately improving spatial resolution and compatibility with minimally invasive devices. We selected two representative trace lengths relevant for neural probe applications in rodents, targeting deep brain regions where neurotransmitter detection is commonly performed. The trace widths were chosen starting from 1 μm—the minimum resolution achievable with standard photolithography—to reflect the lower limit of feasible interconnect dimensions. Building on the sheet resistance results (see Table 1), we extend the analysis of GC by examining the effects of trace dimensions on their electrical resistance *R* (Figure 1) that is calculated using the following equation:

$$R=\rho_{s}\frac{l}{w}$$ (1)

In the Equation 1, $\rho_{s}$ is the measured sheet resistance (Ω/square), l is the trace length (m), and w is the trace width ( m ). (Nam et al., 2022; Wellman et al., 2018).

We observe that increasing the trace width and GC thickness leads to lower resistance values, as shown in Figures 1a,b. For comparison, resistance was calculated for the Cr/Au/Pt metal stack with a total thickness of 120 nm used for ultra-flexible nanoelectronic probes (Luan et al., 2017; Wei et al., 2018; Zhao et al., 2023). Metal traces exhibited significantly lower resistance than GC traces, particularly at smaller widths (1–3 µm). Specifically, to achieve resistance values on the same order of magnitude, GC traces must be approximately ten times wider and around 31 times thicker than their metal counterparts. However, as shown in Figures 1a,b, when the trace width reaches approximately 10 μm, GC interconnect resistance approaches that of metals. This difference in resistance can have direct implications for device operation. In neural interfaces, the cross-sectional dimensions of a trace dictate the maximum signal or stimulation current that can be delivered without excessive Joule heating (P), given by

$$P=I^{2}R$$ (2)

In the Equation 2, *I* is the electrical current (A) and *R* is the resistance (Ω). (Wellman et al., 2018).

Low-resistivity metals can carry high currents through narrow traces with minimal heating, while the higher resistivity of carbon materials necessitates larger lateral dimensions or cross-sectional area to maintain safe thermal limits. However, electrical conductivity alone does not determine performance. Thin-film metals may easily corrode, dissolve, or delaminate under prolonged stimulation (Wellman et al., 2018; Tintelott et al., 2022; Lecomte et al., 2018; Vomero et al., 2017), such as constant FSCV waveform application, while GC offers exceptional electrochemical stability, a wider water electrolysis window, and higher charge injection capacity without harmful reactions (Nimbalkar et al., 2018; Vomero et al., 2017). These properties make GC and other carbon materials advantageous for electrochemical applications, where long-term stability and safe stimulation are imperative and outweigh the need for the lowest possible resistance. In such cases, a modest increase in GC cross section is a worthwhile trade-off, especially considering the corrosion risks of metal films, even with protective adhesion layers. Please refer to the FSCV stability results discussed in dept in Section 3.4.

Based on this analysis and our proof of concept (Faul et al., 2024), GC enables a level of miniaturization that exceeds the current limits of LIG technology. A notable LIG-based example is NeuroStrings—graphene electrodes decorated with Fe_3_O_4_ nanoparticles for FSCV sensing in neural implants—fabricated using a 6 W laser to form a 50–80 μm-thick graphene nanofiber layer with ∼100 μm trace widths (Li et al., 2022). In contrast, GC-based interconnects enable far smaller feature sizes, highlighting their feasibility and advantages in miniaturization despite their lower electrical conductivity compared to ultrathin, flexible metal-based nanoelectronic probes (Luan et al., 2017; Wei et al., 2018; Zhao et al., 2023). In our previous work, “all”-GC MEAs with 3 μm-wide, 2 μm-thick, 5 mm-long GC traces exhibited electrochemical impedance comparable to hybrid GC-MEAs (metal traces and GC electrodes) and successfully detected 5-HT using FSCV (Faul et al., 2024), showing that the high conductivity provided by materials such as gold and platinum may not be strictly necessary to ensure adequate functional and sensing performance.

### 3.2 Electrical properties of double pyrolyzed glassy carbon

To enable the miniaturization of the “all”-GC-MEAs and increase channel counts within a reduced device footprint, one promising strategy is to adopt a double-layer fabrication approach, wherein electrode traces are distributed across multiple layers to support the development of high-density arrays. To date, such a process has not been demonstrated for implantable devices using GC electrodes and interconnects, but it would allow for higher channel counts while maintaining miniaturized shank dimensions. A critical prerequisite is verifying that GC can undergo a second pyrolysis without degradation. For the double-layer fabrication approach to be feasible, GC must retain the electrical, morphological, and surface properties essential for reliable electrochemical sensing after the second pyrolysis.

Although numerous studies have investigated the effects of varying pyrolysis temperatures on the properties of GC (Jurkiewicz et al.; Yang et al., 2023; Yang, 2024; Sharma et al., 2018), to the best of our knowledge, this work is the first to examine the structural, electrical and sensing properties of GC subjected to two consecutive pyrolysis cycles, providing critical insight into the impact of repeated thermal processing on the structural and electrical properties of GC- MEAs. The same GC samples with the three previously analyzed thicknesses underwent a second pyrolysis process under identical conditions to the initial cycle. Following this, four-point probe measurements were repeated as previously described to assess the impact of the second pyrolysis on GC conductivity. Table 2 summarizes the sheet resistance measurements of GC samples with three different thicknesses, after the second pyrolysis cycle, and compares them with metal samples of the same dimensions. After the second pyrolysis, additional shrinkage was observed across all thicknesses. Specifically, the thickest GC shrank from 3.81 μm to 2.99 μm exhibiting a 21.6% reduction in thickness, whereas the intermediate shrank from 2.81 μm to 2.31 μm exhibiting a 17.8% decrease, and the thinnest shrank from 1.98 μm to 1.62 μm exhibiting an 18.0% decrease, respectively. On average, the second pyrolysis generates an additional shrinkage of 0.17% in length and 0.76% in width. Supplementary Figure 1 shows optical images of electrodes and traces after the first and second pyrolysis.

**TABLE 2:** Sheet resistance of double-pyrolyzed glassy carbon at three thicknesses compared to metal.

Material	Thickness (µm) (*n* = 10)	Sheet resistance *ρ_s_* (Ω/square) (*n* = 14)
GC	2.99 ± 0.077	96.22 ± 1.24
GC	2.31 ± 0.060	113.94 ± 4.17
GC	1.62 ± 0.026	150.38 ± 2.84
Metal (Cr, Au, Pt)	0.12	2.44 ± 0.39

The observed increase in sheet resistance after the second pyrolysis cycle arises from combined dimensional and microstructural changes in the GC. Prolonged high-temperature exposure—2 h at 900 °C plus an additional ∼2 h between 800 °C and 900 °C—drives volumetric contraction and structural densification, reducing the effective film thickness (Uskoković, 2021). Given that sheet resistance is inversely proportional to thickness, this shrinkage directly increases resistance. Concurrently, the second thermal treatment induces structural reorganization within the GC matrix, lowering electron density and further elevating resistance (Uskoković, 2021). Raman analysis, discussed in detail in Section 3.3, together with XRD results, confirms that densification is accompanied by increased structural disorder and diminished graphitic order (Pramanick et al., 2018; Ferrari and Robertson, 2000). Fragmentation of extended sp^2^ domains during the second pyrolysis cycle disrupts the continuity of π-electron pathways, thereby contributing to the observed increase in electrical resistance (Yang et al., 2023; Ferrari and Robertson, 2000).

**FIGURE 2:**
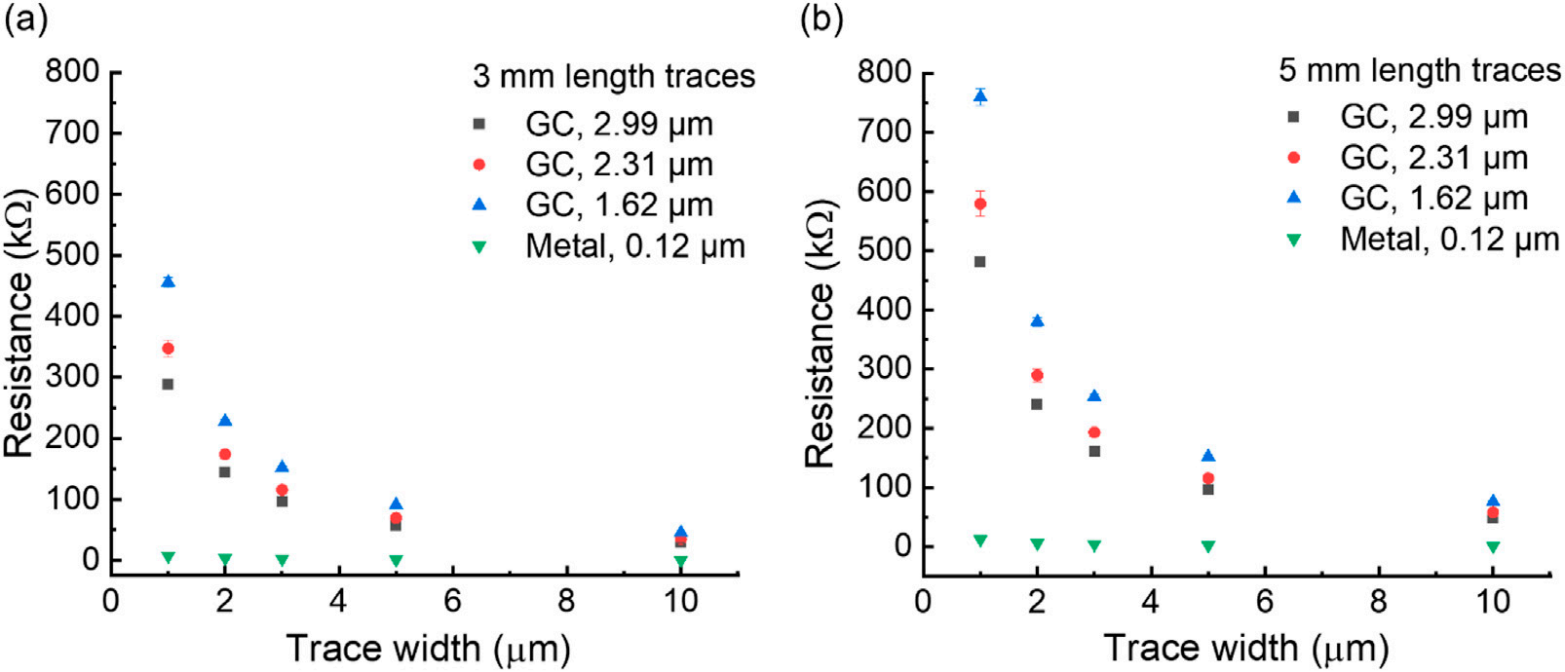
Resistance of pyrolyzed twice GC with respect to trace width for 2.99μm, 2.31 μm, and 1.62 μm thicknesses, for 3 mm length **(a)** and 5 mm length **(b)** traces*,* in comparison with metal traces of the same width and 120 nm thickness (Cr 15 nm, Au 20 nm, Pt 85 nm).

Building on the sheet resistance results, we extend the analysis of twice pyrolyzed GC by examining the effects of the dimensions of the traces (interconnects) on overall resistance, as previously performed for once pyrolyzed GC (Figure 2).

The electrical resistance of GC traces follows the same trend as the sheet resistance, showing an average increase of approximately 88% after the second pyrolysis compared with single-pyrolyzed GC traces. This increase also accounts for material shrinkage resulting from the second pyrolysis step. Detailed resistance values are provided in Supplementary Tables 1, 2. Based on these values, achieving equivalent resistance would require double-pyrolyzed GC traces to be ∼88% wider than single-pyrolyzed traces of equal thickness (approximately 9 µm *versus* 5 µm), while remaining much smaller than what is currently achievable with LIG.

This finding highlights a necessary design trade-off between GC trace width (or cross-sectional area) and electrical resistance, which is critical for guiding the design of neural probes with optimized electrode and interconnect dimensions. For example, at a given GC thickness, a 5-mm-long trace with a width of 10 µm exhibits resistance comparable to that of a 1-µm-wide metal trace (see Supplementary Table 2). Thus, despite the higher resistance, the required increase in GC trace size remains practical and within acceptable limits.

Importantly, this trade-off is justified by the superior electrochemical stability of GC compared with metals. Moreover, previous FSCV studies using carbon traces (Faul et al., 2024; Li et al., 2022; Nam et al., 2022) suggest that the extremely high conductivity of materials such as gold or platinum may not be strictly necessary to achieve adequate functional and sensing performance, as discussed in the previous section.

Although double-pyrolyzed GC exhibits less favorable electrical properties, it enables the fabrication of multi-layer “all-GC” MEAs with interconnects distributed across different layers. Such an architecture could significantly increase electrode packing densities in future “all-GC” MEAs, making the size trade-off worthwhile.

In addition to considerations regarding conductor and sensor materials, ensuring the reliability of double-layer “all”-GC-MEA fabrication requires integrating a suitable insulating material that can not only maintain electrical isolation but also provide structural stability between layers and withstand the high temperatures associated with the pyrolysis process.

Our previous work has primarily relied on flexible polymeric insulators such as SU-8 and polyimide (Vahidi et al., 2020; Vomero et al., 2017; Yang et al., 2024; Wu et al., 2024; Wu et al., 2023; Salavatian et al., 2023) due to their mechanical compatibility with brain tissue and ability to reduce chronic inflammation (Nimbalkar et al., 2018; Agorelius et al., 2015; Castagnola et al., 2013). However, these materials are unsuitable for multilayer GC-MEA designs that require processing temperatures exceeding 900 °C. To enable double-layer fabrication in future work, we will explore the use of silicon nitride (Si_3_N_4_), which is not only compatible with high-temperature pyrolysis but also possesses essential properties for chronic neural interfaces—including biocompatibility, chemical resistance, mechanical robustness, and excellent electrical insulation (HajjHassan et al., 2008; Wise et al., 2004; Shen and Maharbiz, 2021; Kim et al., 2021). Si_3_N_4_ has long been employed as an insulating substrate for microfabricated neural probes (HajjHassan et al., 2008; Wise et al., 2004; Shen and Maharbiz, 2021; Yi et al., 2022). Although its elastic modulus, in the hundreds of GPa (Kim et al., 2021; Zerr et al., 2002), is significantly higher than that of polymeric substrates (Castagnola et al., 2023b), the fabrication of ultrathin semiconductor membranes allows silicon-based electronics to achieve remarkable flexibility (Liu et al., 2016; Viventi et al., 2011). Compared to alternatives such as silicon dioxide and silicon carbide (SiC), Si_3_N_4_ offers superior mechanical strength, lower water permeability, enhanced chemical resistance, and better compatibility with established microfabrication workflows (Pezzotti and McEntire, 2024). While amorphous SiC (a-SiC) has also shown promise as an insulating coating (Cogan et al., 2003; Deku et al., 2018), it may not withstand the thermal requirements of GC pyrolysis. Hexagonal boron nitride (h-BN) will also be considered as a potential alternative due to its dielectric stability, chemical resistance, and compatibility with carbon-based electronics (Kim et al.; Ashton and Moore, 2017; Ngamprapawat et al., 2023).

A hybrid material approach may also be consider to reduce overall device stiffness, such as additional encapsulation layers using soft, patternable dielectrics—such as photo patternable polyimide or KMSF^®^ 1000 (Vomero et al., 2017; Zheng et al., 2021). Prior studies have shown that ultrathin silicon membranes integrated onto soft substrates can achieve high flexibility and mechanical compliance (Liu et al., 2016; Seo et al., 2016; Zhao et al., 2018). Furthermore, we recently developed a double-etching process to fabricate flexible GC fibers using a thin Si_3_N_4_ bottom layer and SU-8 top insulation (Siwakoti et al., 2025a). The SEM and the EDS, confirmed the structural integrity of the design and compositional uniformity of the Si_3_N_4_ insulating layer (Siwakoti et al., 2025a). These results support the feasibility of a hybrid material approach, for creating compliant, high-performance microelectrodes. While the polymeric substrate provides intimate, conformal contact with curved biological surfaces, the silicon layer enhances mechanical rigidity to support device insertion (Sim et al., 2018), (Siwakoti et al., 2025a).

### 3.3 Structural and morphological characterization of single and double pyrolyzed glassy carbon

In addition to investigating the electrical properties of GC, it is essential to characterize the structural and morphological differences between single- and double-pyrolyzed GC. Raman spectroscopy and XRD were employed to assess the degree of graphitization, defect density, and crystallinity of the GC surfaces.

**FIGURE 3:**
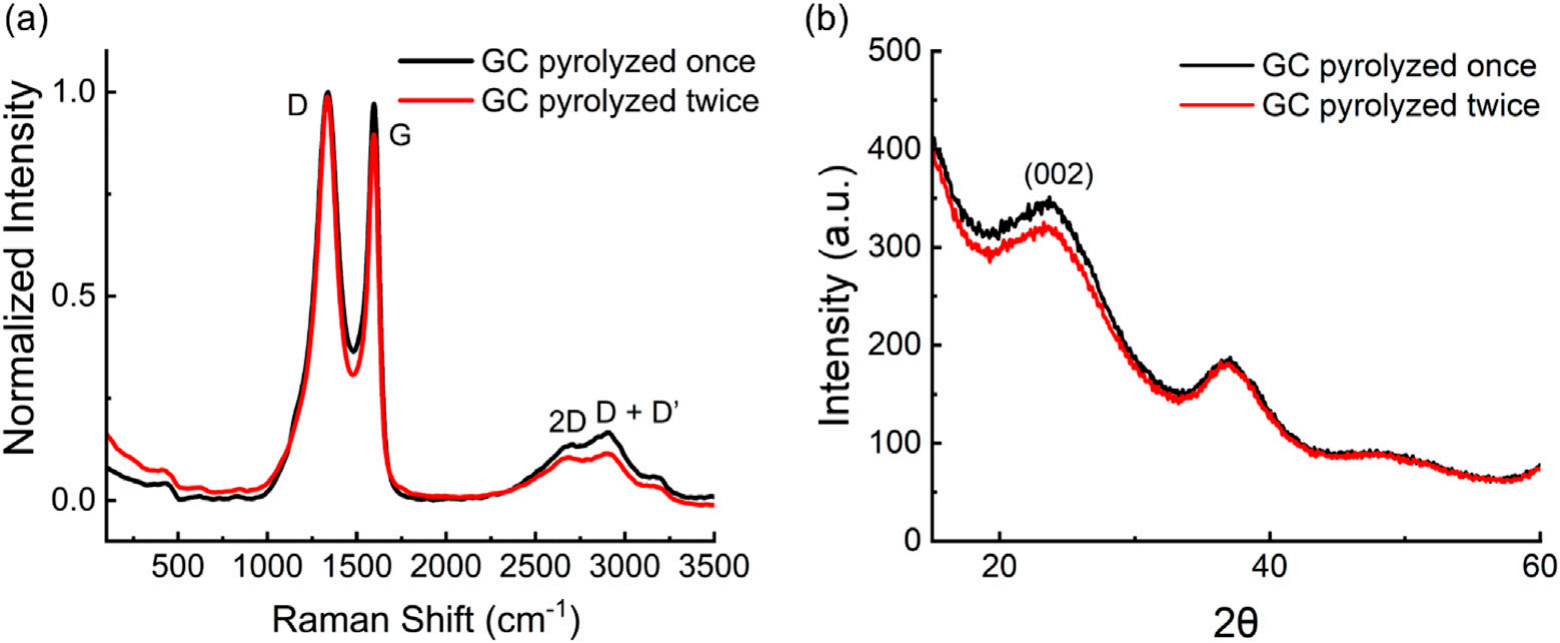
**(a)** Raman spectra of GC electrodes fabricated using one (black) and two (red) pyrolysis cycles; **(b)** XRD spectra of GC electrodes fabricated using one (black) and two (red) pyrolysis cycles.

Representative Raman spectra of GC obtained after one and two pyrolysis cycles are reported in comparison in Figure 3a. Raman spectra of both single- and double-pyrolyzed GC microelectrodes exhibited the characteristic structural features of carbon-based materials, with prominent D and G in the typical range of 1200–1700 cm^-1^. The D peak at 1340 cm^-1^ corresponds to disorder in the graphite structure, while the G peak at 1600 cm^-1^ represents ordered sp^2^ graphitic carbon (Castagnola et al., 2024; Uskoković, 2021). The positions of these peaks were consistent across both fabrication methods. However, the D/G intensity ratio, used to evaluate the defect level of carbon materials, differed between groups (Table 3).

**TABLE 3:** D/G ratio values of GC pyrolyzed once and twice.

D/G ratio Pyrolyzed once (mean ± sd, *n* = 6)	D/G ratio Pyrolyzed twice (mean ± sd, *n* = 6)
1.008 ± 0.027	1.057 ± 0.017

An unpaired two-sample t-test revealed a statistically significant increase in the D/G ratio after the second pyrolysis (*p* = 0.0042).

Single-pyrolyzed GC had a mean D/G ratio of 1.008 ± 0.027, indicating a more ordered carbon structure, whereas double-pyrolyzed GC had a ratio of 1.057 ± 0.017, suggesting increased structural disorder (Uskoković, 2021). This change resulted primarily from a decrease in G band intensity, while the D band remained nearly unchanged. The stability of the D band suggests that no significant new sp^3^-type defects or edge-plane sites were generated, while the decrease in the G band indicates fragmentation of extended sp^2^ domains (Ferrari and Robertson, 2000).

Both single- and double-pyrolyzed GC also exhibited broader second-order Raman features between 2500 and 3500 cm^-1^, including the 2D band at 2700 cm^-1^ and the D + D′ band at 2900 cm^-1^. These features confirm the presence of partially graphitized carbon with some degree of layered organization (Castagnola et al., 2024; Ferrari and Basko, 2013). The lower intensity of the 2D and D + D′ peaks after the second pyrolysis suggests reduced crystallinity compared with single-pyrolyzed GC (Tyler et al.).

XRD analysis aligns with the findings from Raman spectroscopy. Single-pyrolyzed GC exhibited a prominent peak near 23° (2θ), corresponding to the (002) plane, indicative of some stacking between carbon layers and higher local order (Figure 3b). This peak weakened after the second pyrolysis, reflecting reduced graphitic stacking and structural alignment (Tyumentsev and Fazlitdinova, 2020; Xu et al., 2024). These changes did not produce a notable increase in oxygen-containing functional groups or electrochemically active sites, consistent with the sensitivity data in Section 3.4. This suggests that the carbon microstructure reaches thermal saturation at 900 °C, where extended heat treatment favors amorphization over further graphitization (Sharma et al., 2018; Ferrari and Robertson, 2000; Franklin, 1951).

Finally, the SEM-EDS confirmed that the elemental atomic percentages of GC remained unchanged after the second pyrolysis. No statistically significant differences were detected in the oxygen or carbon atomic percentages (Table 4), indicating that repeated pyrolysis did not substantially alter the elemental composition.

**TABLE 4:** Elemental atomic percentage of GC samples after single and double pyrolysis, obtained using Scanning Electron Microscopy coupled with Energy-Dispersive X-ray Spectroscopy (SEM-EDS).

Atomic %	Pyrolyzed once (mean ± sd, *n* = 6)	Pyrolyzed twice (mean ± sd, *n* = 6)
Carbon	92.715 ± 0.252	92.272 ± 1.229
Oxygen	7.285 ± 0.251	7.728 ± 1.229

### 3.4 Fast scan cyclic voltammetry detection

To evaluate the effect of a second pyrolysis cycle on the sensitivity of GC microelectrodes for the detection of 5-HT and DA—two key neurotransmitters involved in mood regulation, cognition, and various physiological functions—we compared the FSCV performance of the GC microelectrodes of hybrid GC-MEAs fabricated using single and double pyrolysis processes. FSCV is widely regarded as the gold standard electrochemical technique for detecting electroactive neurotransmitters, relying on direct electron transfer between redox-active molecules and the carbon electrode surface (Rafi and Zestos, 2021; Roberts and Sombers, 2018; Siwakoti et al., 2025b; Venton and Cao, 2020; Meunier and Sombers, 2021). By applying a rapid potential sweep (400–1200 V/s) at a repetition frequency of 10 Hz, FSCV provides sub-second temporal resolution (Rafi and Zestos, 2021; Roberts and Sombers, 2018; Siwakoti et al., 2025b; Venton and Cao, 2020; Meunier and Sombers, 2021). Various FSCV waveforms have been optimized for different analytes, including 5-HT, dopamine (DA), adenosine (AD), melatonin (MT), to maximize sensitivity and reduce electrode fouling (Takmakov et al., 2010; Nguyen and Venton, 2015; Castagnola et al., 2020; Hensley et al., 2018; Jackson et al., 1995; Heien et al., 2003; Dunham and Venton, 2020).

In this study, FSCV measurements were conducted on hybrid GC-MEAs subjected to both single and double pyrolysis to assess sensitivity toward 5-HT and DA detection. Each electrode was tested using a 1 μM bolus injection of 5-HT and DA, respectively. For 5-HT detection, a modified N-shaped Jackson waveform (0.2 V → 1.3 V → −0.1 V → 0.2 V vs. Ag/AgCl) was applied at a scan rate of 1000 V/s (Jackson et al., 1995). This waveform was selected for its demonstrated ability to minimize electrode fouling, which is especially critical given the high susceptibility of 5-HT to fouling (Faul et al., 2024; Jackson et al., 1995; Dunham and Venton, 2020). For DA detection, a triangular waveform (−0.4 V → 1.0 V → −0.4 V vs. Ag/AgCl) was used at a scan rate of 400 V/s, consistent with previously reported protocols (Castagnola et al., 2021).

**FIGURE 4:**
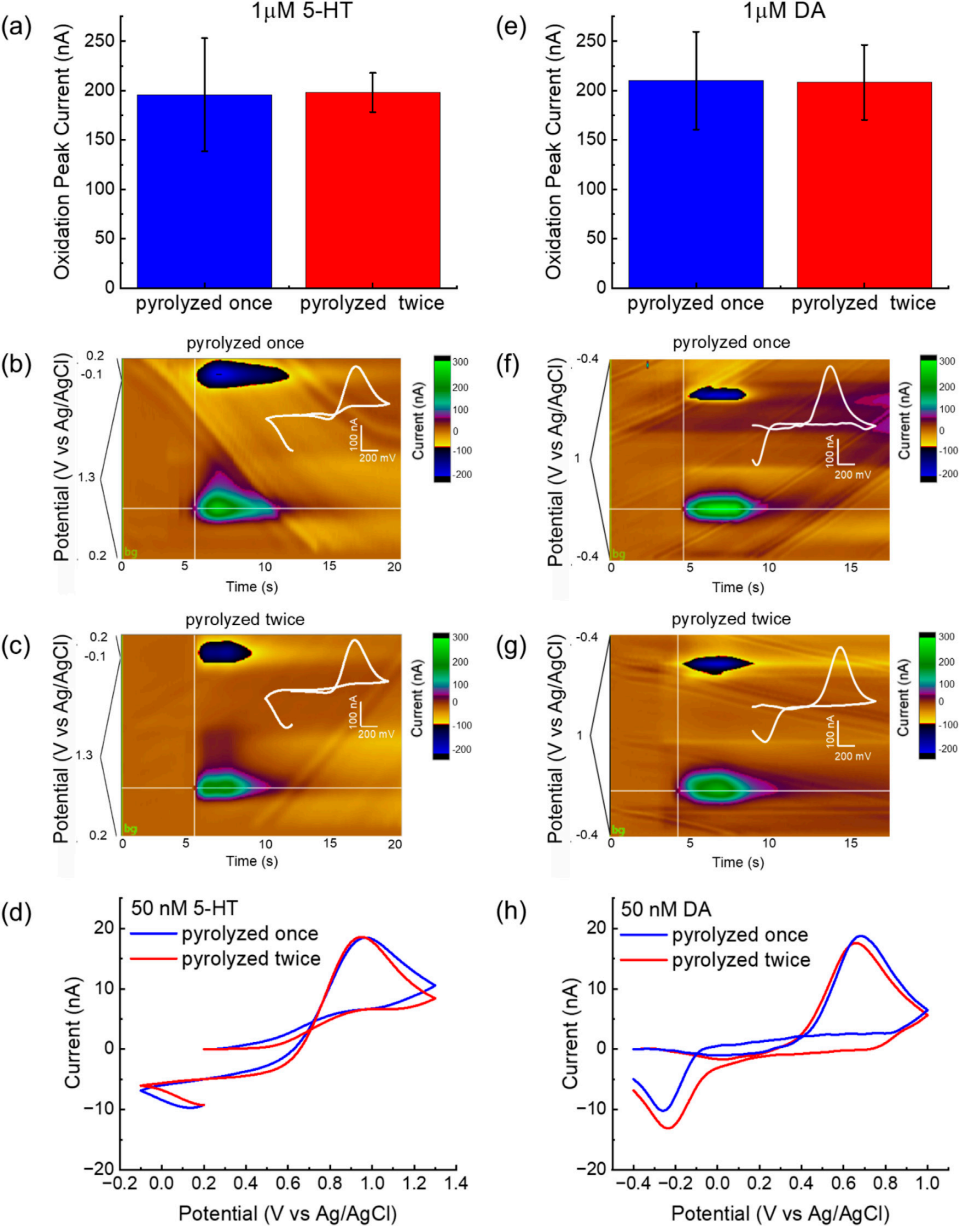
Comparison of FSCV detection performance for 5-HT and DA using single- and double-pyrolyzed GC microelectrodes. **(a)** Comparison of 5-HT oxidation peak current (mean ± sd, *n* = 12) measured using FSCV for the 2 GC conditions. **(b,c)** Representative color plots and corresponding background-subtracted cyclic voltammograms (inserts) obtained using **(b)** single-pyrolyzed GC and **(c)** double-pyrolyzed GC for 5-HT detection. **(d)** Representative background-subtracted cyclic voltammograms in response to 50 nM 5-HT bolus injections, collected using single-pyrolyzed GC (blue) and double-pyrolyzed GC (red). **(e)** Comparison of DA oxidation peak current (mean ± sd, *n* = 18) measured using FSCV for the 2 GC conditions. **(f,g)** Representative color plots and corresponding background-subtracted cyclic voltammograms (inserts) obtained using **(f)** single-pyrolyzed GC and **(g)** double-pyrolyzed GC for DA detection. **(h)** Representative background-subtracted cyclic voltammograms in response to 50 nM DA bolus injections, collected using single-pyrolyzed GC (blue) and double-pyrolyzed GC (red).

Both single- and double-pyrolyzed GC microelectrodes exhibited robust and reproducible oxidation responses to 1 μM 5-HT and DA. The oxidation peak currents recorded during FSCV for the two fabrication methods are shown in Figure 4a for 5-HT and Figure 4e for DA. Statistical analysis using one-way ANOVA followed by Bonferroni *post hoc* tests revealed no significant differences in DA and 5-HT peak current amplitudes between electrodes fabricated with single *versus* double pyrolysis, indicating similar electrochemical sensitivity. These findings align with the previously discussed Raman spectroscopy and XRD results, suggesting that the second pyrolysis cycle introduces structural disorder without necessarily creating additional edge-plane sites or electroactive functionalities (Ferrari and Robertson, 2000) (Franklin, 1951). Interestingly, although mean sensitivity remained statistically unchanged, the standard deviation of FSCV responses across electrodes decreased noticeably, indicating improved consistency and uniformity in its electrochemical performance across multiple measurements and microelectrodes. The relative standard deviation, calculated as the ratio of the standard deviation to the mean and expressed as a percentage, dropped from 29% to 10% for 5-HT, and from 24% to 18% for DA. This improvement likely reflects thermal stabilization during the second pyrolysis, which may reduce residual stress, surface roughness variability, and shrinkage inconsistencies—factors known to affect electrochemical reproducibility in microfabricated carbon electrodes (Hassan et al., 2017). This enhanced consistency could contribute to better reliability and reduced calibration requirements in sensing applications. Figures 4b,c show representative color plots of FSCV responses to a 1 μM bolus injection of 5-HT for GC microelectrodes fabricated using single and double pyrolysis cycles, respectively. Similarly, Figures 4f,g display representative color plots of FSCV responses to a 1 μM bolus injection of DA for electrodes fabricated with single and double pyrolysis, respectively. In FSCV, color plots provide a three-dimensional representation of electrochemical data, where the x-axis denotes time, the y-axis represents the applied voltage, and the current response is encoded using a color scale. In these plots, oxidation and reduction events appear as distinct color changes—typically warm colors (e.g., green) for oxidation peaks and cool colors (e.g., blue) for reduction peaks. Distinct oxidation and reduction peaks related to the redox activity of 5-HT (Figures 4b,c) and DA (Figures 4f,g) are clearly visible in the pseudo-color plots for both single- and double-pyrolyzed electrodes. Specifically, 5-HT shows oxidation at approximately 0.9 V and reduction at 0.2 V *versus* Ag/AgCl, while DA exhibits oxidation at around 0.6 V and reduction at −0.25 V *versus* Ag/AgCl. These color features appear immediately following the injection of the 1 μM 5-HT or DA bolus via the flow cell system. The signals gradually dissipate as the analytes are cleared from the electrode surface by a continuous flow of 1× PBS, delivered through the flow cell using a syringe pump. These electrochemical oxidation-reduction features are further supported by oxidation and reduction peaks in the background-subtracted cyclic voltammograms shown in the insets of Figures 4b,c, for 5-HT, and Figures 4f,g, for DA.

Based on ours previous findings (Castagnola et al., 2022; Siwakoti et al., 2025a; Robbi et al., 2022) and work by others (Robinson et al., 2014; Wu et al., 2001), phasic DA release has been detected at concentrations ranging from several hundreds of nanomolar to several micromolar following drug administrations. Phasic 5-HT release has been observed between 30 and 50 nM and several hundreds of nM in different brain regions before and after pharmacological manipulation (Abdalla et al., 2020; Mena et al., 2024; Saylor et al., 2019; Yuen et al., 2022; Wood and Hashemi, 2013).

To demonstrate that our GC microelectrodes can detect analytes within the physiological concentration ranges of DA and 5-HT—which may fall below 1 μM, particularly for 5-HT—we detected 50 nM bolus injections of both neurotransmitters using GC microelectrodes fabricated via single and double pyrolysis, as reported in Figure 4d (for 5-HT) and Figure 4h (for DA). Both electrode types reliably detected physiologically relevant concentrations, consistent with our prior *in vivo* results using single-pyrolysis GC (Castagnola et al., 2022; Castagnola et al., 2024; Siwakoti et al., 2025a).

The theoretical limits of detection (LOD), calculated as three times the standard deviation of the noise, were 1.5 nM (single pyrolysis) and 2.2 nM (double pyrolysis) for 5-HT, and 1.1 nM (single) and 1.5 nM (double) for DA, showing a slight increase in LOD with double pyrolysis. These values remain comparable to previously reported results obtained with GC microelectrodes (Castagnola et al., 2021; Siwakoti et al., 2025a).

**FIGURE 5:**
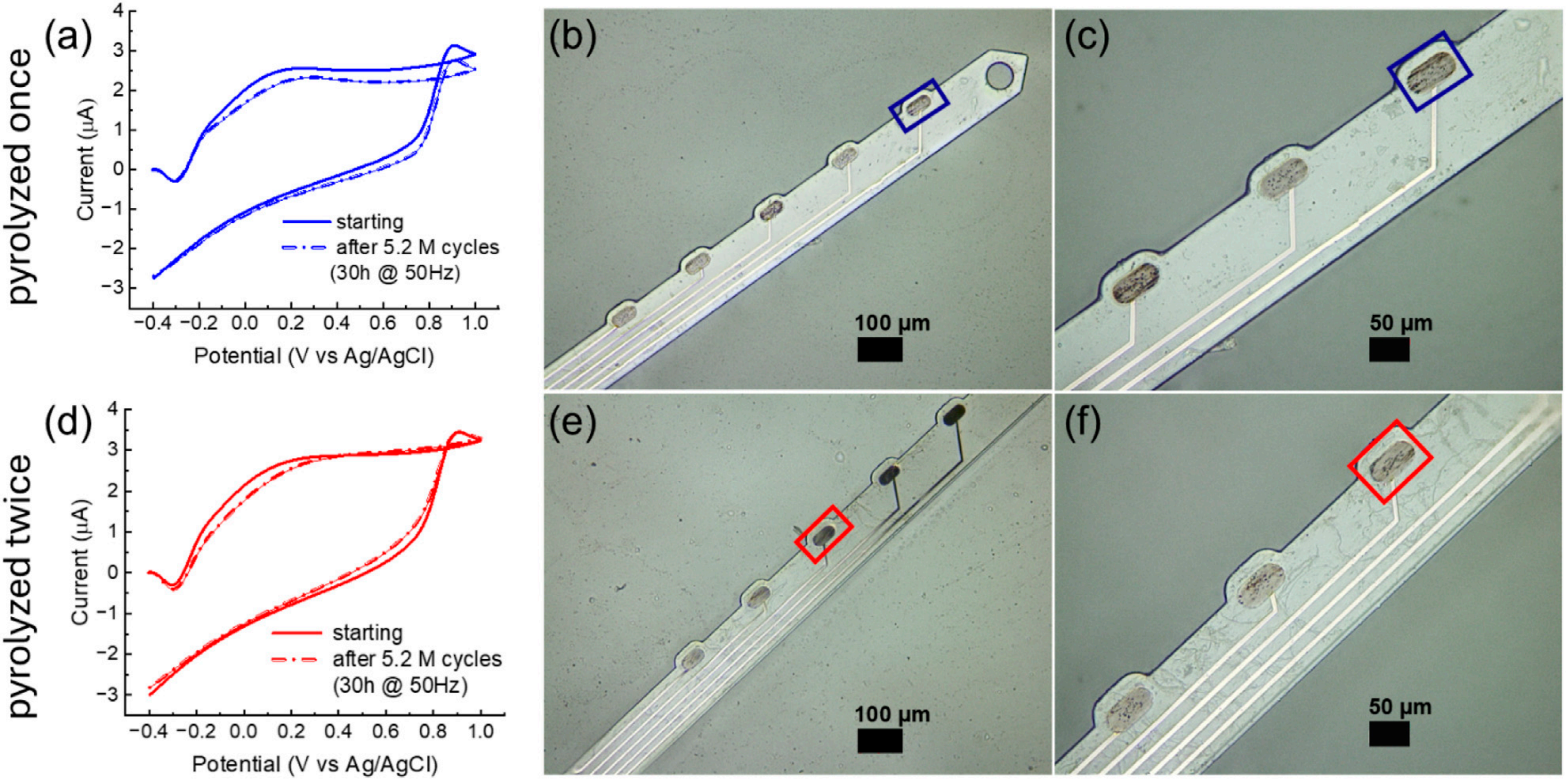
**(a)** Background current of a GC microelectrode obtained with a single pyrolysis step, measured before and after application of the FSCV waveform used for DA detection (−0.4–1.0 V *versus* Ag/AgCl, 400 V/s) at 50 Hz for 30 consecutive hours. **(b,c)** Optical image of a hybrid GC-MEA shank with a magnified view of the stimulated electrodes (single pyrolysis, outlined in blue) *versus* non-stimulated electrodes. **(d)** Background current of a GC microelectrode obtained with double pyrolysis. **(e,f)** Optical image of a hybrid GC-MEA shank with a magnified view of the stimulated electrodes (double pyrolysis, outlined in red) *versus* non-stimulated electrodes.

To assess the stability of the background current over time, we applied the FSCV waveform used for DA detection (−0.4–1.0 V *versus* Ag/AgCl, 400 V/s) at 50 Hz to GC microelectrodes pyrolyzed once or twice in PBS for 30 consecutive hours. Prior to testing, the electrodes were preconditioned by cycling at 60 Hz for 30 min using the same waveform, similar to preconditioning protocols reported for carbon fibers (Takmakov et al., 2010; Meunier et al., 2017) and LIG (Li et al., 2022). Both single- and double-pyrolyzed GC microelectrodes maintained stable background currents over 30 h of continuous FSCV cycling at 50 Hz, showing only minor current variations twice 8.9% and 2.3% once (Figure 5) with an approximately 3%lateral etching of the stim GC microelectrodes.

Both single- and double-pyrolyzed GC microelectrodes maintained relatively stable background currents during 30 h of continuous FSCV cycling at 50 Hz, exhibiting only minor current variations (8.9% for single-pyrolyzed and 2.3% for double-pyrolyzed, as shown in Figures 5a,d). Optical images (Figures 5b,c for single-pyrolyzed and Figures 5e,f for double-pyrolyzed) show no evident etching of the stimulated GC microelectrodes. In contrast, metal electrodes degraded within 75 min under the same conditions (Supplementary Figure 2).

The background currents of our GC microelectrodes—both single- and double-pyrolyzed—is approximately six times higher than that reported for LIG graphene electrodes decorated with Fe_3_O_4_ nanoparticles, graphene with NiO nanoparticles (Li et al., 2022), and two to three times higher than commercial carbon fibers (Li et al., 2022; Castagnola et al., 2020; Robbi et al., 2022). When normalized by geometric area, the background currents of both single- and double-pyrolyzed GC microelectrodes are comparable to those of heteroatom-doped LIG porous graphene (N-doped, F-doped, and S-doped) (Nam et al., 2022).

Overall, the second pyrolysis cycle does not negatively affect the sensing capabilities of GC, reinforcing the potential of a double-layer fabrication process for higher-density “all”-GC-MEAs.

## 4 Conclusion

This study presents a comprehensive analysis of how GC thickness, trace width, and trace length influence the electrical properties of GC fabricated using one and two pyrolysis cycles, with a focus on the fabrication and miniaturization of the next-generation of “all”-GC-MEAs, selecting appropriate electrode and interconnect dimensions to ensure reliable performance. Double pyrolysis of GC traces resulted in ∼20% dimensional shrinkage across all initial thicknesses, leading to an ∼88% increase in sheet resistance on average. To assess the miniaturization potential of GC interconnects, we examined the effect of trace dimensions on electrical resistance. As expected, increasing both trace width and GC thickness led to reduced resistance. For comparison, resistance values were benchmarked against Cr/Au/Pt metal traces (total thickness 120 nm), commonly used in ultra-flexible nanoelectronic probes. Metal traces exhibited significantly lower resistance than GC, especially at narrower widths (1–3 µm), where GC traces must be approximately ten times wider and 31 times thicker to reach comparable resistance levels. However, when the trace width reaches 5–10 μm, the resistance of GC interconnects approaches that of metal traces. These findings demonstrate the feasibility of using GC as a viable material for miniaturized interconnects in neural interfaces, offering substantial advantages over LIG-based technologies in terms of miniaturization, while highlighting the design trade-offs necessary to approach the electrical performance of ultrathin metal-based nanoelectronic probes. Although GC exhibits higher sheet resistance than noble metals, it provides unique benefits for neurochemical sensing and superior electrochemical stability, supporting its use in multimodal neural applications.

GC produced with one or two pyrolysis cycles was characterized by Raman, XRD, elemental analysis, and FSCV for 5-HT and DA detection. A second cycle preserved structural integrity and sensing performance, supporting its use in double-layer “all”-GC-MEA fabrication.

Collectively, these findings deepen our understanding of GC’s electrical and sensing properties, guiding optimization of batch-fabricated, high-density “all”-GC-MEAs. To the best of our knowledge, this work is the first to examine the electrical and morphological properties of GC subjected to two consecutive pyrolysis cycles. It also offers a detailed examination of GC’s electrical and morphological behavior at highly miniaturized scales, further advancing its suitability for neural probe applications.

## Data Availability

The original contributions presented in the study are included in the article/Supplementary Material, further inquiries can be directed to the corresponding author.
